# Protective Effects of Estrogen on Cardiovascular Disease Mediated by Oxidative Stress

**DOI:** 10.1155/2021/5523516

**Published:** 2021-06-28

**Authors:** Du Xiang, Yang Liu, Shujun Zhou, Encheng Zhou, Yanfeng Wang

**Affiliations:** Zhongnan Hospital of Wuhan University, Institute of Hepatobiliary Diseases of Wuhan University, Transplant Center of Wuhan University, Wuhan 430071, China

## Abstract

Perimenopause is an important stage of female senescence. Epidemiological investigation has shown that the incidence of cardiovascular disease in premenopausal women is lower than that in men, and the incidence of cardiovascular disease in postmenopausal women is significantly higher than that in men. This phenomenon reveals that estrogen has a definite protective effect on the cardiovascular system. In the cardiovascular system, oxidative stress is considered important in the pathogenesis of atherosclerosis, myocardial dysfunction, cardiac hypertrophy, heart failure, and myocardial ischemia. From the perspective of oxidative stress, estrogen plays a regulatory role in the cardiovascular system through the estrogen receptor, providing strategies for the treatment of menopausal women with cardiovascular diseases.

## 1. Introduction

Cardiovascular disease (CVD) has the highest mortality in the world [1]. With the aging of the population and the increasing incidence of obesity and diabetes, the cost of treatment for CVD will significantly increase worldwide [2]. The incidence of CVD is related to gender, and premenopausal women have a lower incidence of hypertension, atherosclerosis, myocardial dysfunction, ventricular hypertrophy, heart failure, and myocardial ischemia than age-matched men [3]. However, the advantage in women gradually disappears after menopause, which leads to a higher risk of CVD in postmenopausal women than men of the same age. This trend is largely attributed to the role of female estrogen in this process [4]. During the transitional period of menopause, women suffer from blood vessel aging, decreased diastolic ability, insulin sensitivity, and increased blood pressure due to decreased ovarian function and changes in hormone secretion, which increase the risk of CVD development [5]. Several studies have shown that certain functions mediated by estrogen in the cardiovascular system are related to the reduction in local oxidative stress (OS), which can reduce reactive oxygen species (ROS) by regulating the production of ROS enzymes and can enhance ROS clearance [6].

Estrogen has a wide range of critical physiological effects and exerts crucial effects on the growth and maturation of the endocrine, cardiovascular, skeletal, and metabolic systems [7]. With the extension of the human life span, the population of China is gradually aging; so, women will live nearly one-third of their lives without estrogen protection [8]. The decline in the ovarian function and the reduction in estrogen during menopause usually result in physical and psychological changes in females and lead to a series of autonomic dysfunction symptoms (sweating, irritability, insomnia, hot flashes, etc.) [9]. In addition, heart and brain vascular diseases, osteoporosis, and low immunity, which are related to menopause, have become the main risk factors affecting women's quality of life and life span [10].

Cells are involved in a variety of oxidation reactions in physiological processes, which inevitably leads to the release of ROS and reactive nitrogen species (RNS) [11]. If the balance between ROS and the antioxidant defense mechanism is broken, the accumulated ROS thereby destroy cell macromolecules, cause cell dysfunction, and ultimately kill cells [12]. In the cardiovascular system, excessive ROS production is considered one of the pathogenic mechanisms of atherosclerosis, myocardial dysfunction, myocardial hypertrophy, heart failure, and myocardial ischemia [13]. Reducing the accumulation of ROS in cells, therefore, is a potential strategy to prevent and treat CVD [14]. Estrogen and the body's antioxidant ability decreases as menopausal women grow older, while the body's nicotinamide adenine dinucleotide phosphate (NADPH) and other oxidase activities increase, which results in an inability to clear ROS in time [15]. The accumulated ROS then induce OS, leading to osteoporosis and CVD [16]. Nevertheless, the specific mechanism of how estrogen alleviates CVD remains unclear. This article mainly summarizes the protective effects of estrogen on the cardiovascular system and its mechanism from the perspective of OS, laying the foundation for the treatment of cardiovascular disease in menopausal women.

## 2. Estrogen

Estrogen is a fat-soluble steroid hormone and plays an essential role in the development and physiology of many organ systems, including the breasts, uterus, bone, and cardiovascular system [17]. Estrogen is mainly produced by cholesterol in the ovaries, corpus luteum, and placenta in premenopausal women, with a small amount of estrogen produced by nonovarian organs, such as the liver, heart, skin, and brain [18]. There are three types of estrogen that have been found in the human body: estrone (E_1_), 17*β*-estradiol (E_2_), and estriol (E_3_) [19]. Among these types, E_2_ has the strongest biological activity [20]. E_1_ is synthesized by adrenal dehydroepiandrosterone in the adipose tissue and is more important after menopause; E_2_, the main product of the entire biosynthesis process, is the most effective estrogen before menopause; E_3_, which is produced by E_1_ formed by 16a-hydroxylation, is the weakest estrogen and plays a significant role during pregnancy [21]. In prepubertal women and postmenopausal women, estrogen produced by extragonadal tissues acts locally by paracrine or endocrine means to maintain tissue-specific functions [18]. Estrogen produced by follicles is synthesized by the granulosa cells and inner membrane cells of the follicle under the synergistic effects of follicle-stimulating hormone (FSH) and luteinizing hormone (LH) [18]. Androstenedione and testosterone produced by the inner membrane cells of the follicle under the action of LH diffuse into the granular cells through the basement membrane [22]. Aromatase activity is enhanced under the effects of FSH [23]. Then, androstenedione is converted into estrone, and testosterone is converted into estradiol, which is known as the two-cell-two gonadotropin theory of estrogen synthesis [24]. A small part of the synthesized estrogen enters the follicular cavity, and the majority enter the blood, regulating the differentiation and growth of target cells, such as the endometrium and breasts.

Estrogen inactivation can occur through metabolism, including conversion of E_2_ to less active E_1_ or E_3_ and sulfation by estrogen sulfatase from E_2_ to 17*β*-estradiol-1,3,5-triene-3,17-diol 3-sulfate, so that it no longer interacts with estrogen receptors [25]. In addition, the lack of a new adipose-derived cytokine lipocalin-2 in female mice can limit E_2_ production by downregulating aromatase in the adipose tissue [26]. Therefore, the aromatase that controls the production of estrogen in the body can maintain a dynamic balance between estrogen synthesis and inactivation [27].

## 3. Estrogen Receptor (ER)

The ER is the core target of estrogen to exert its regulatory function and affects diseases in many organ systems including the cardiovascular system and skeletal system [28]. Most human estrogen receptors (ERs) are ligand-dependent transcription factors that belong to the steroid family. Two ERs have been discovered so far: the classic nuclear estrogen receptor (nER) and the membrane estrogen receptor (mER) [29]. The nER has two subtypes: ER*α* and ER*β* [30]. ER*α*, which was discovered by Elwood Jenson in 1958, is widely distributed and has high mRNA expression in the uterus, testes, ovaries, prostate, skeletal muscle, kidneys, skin, etc. [31] In 1996, Kuiper et al. [32] isolated the second nuclear estrogen receptor, ER*β*, which has higher mRNA expression in the ovaries, colon, brain tissue, kidneys, and male reproductive system. With further indepth study of nER, it was found that some target cells can quickly respond to estrogen without ER [33]. Therefore, in addition to the classical nER-mediated slow pathway, there are also fast membrane receptor-mediated estrogen effects that are mediated by G protein-coupled estrogen receptors (GPERs), including G-protein coupled receptor 30 (GPR30) and ER-X [34]. GPR30 is expressed in many brain regions (the hypothalamus, hippocampus, cortex, etc.), the adrenal medulla, renal pelvis, and ovaries [35]. The expression of ER-X is strictly regulated during development, and it is expressed in the brain of fetal baboons and the cerebral cortex, uterus, and lungs of rodents after birth. In adults, ER-X is rarely expressed but is expressed after ischemic injury [36].

## 4. Action Mode of Estrogen

**Figure 1:**
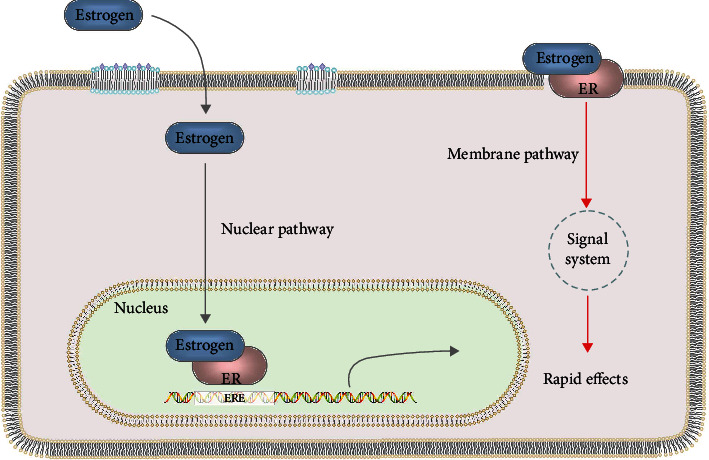
The genomic effect mediated by nuclear ERs and the nongenomic effect mediated by membrane ERs.

The ER structure is mainly divided into five domains: transcription activation region-1 (AF-1), the DNA-binding domain, the ligand-binding domain (LBD), the hinge region, and transcription activation region-2 (AF-2) [37]. Each domain has its specific function, and the LBD is the key area where the ligand recognizes and binds the receptor and then triggers its effects [38]. Most signal pathways mediated by estrogen are regulated by ERs, which can be divided into genomic and nongenomic effects according to whether they are transcriptionally regulated [39]. The classic mode of estrogen action is the genomic effect mechanism in which estrogen enters the nucleus and combines with nuclear ERs to form a dimer, and then the estrogen-receptor complex binds to estrogen response elements and further regulates the gene expression and corresponding proteins, which triggers a series of cascade reaction events [40]. The nongenomic effect does not depend on the gene expression regulation mechanism, and its mode of action is that estrogen binds to the estrogen receptor on the cell membrane and activates the corresponding signal transduction, causing related responses to exert the effects of estrogen [41]. The genomic effect generally works slowly, as it takes several hours to several days to occur, while the nongenomic effect typically only takes a few seconds to a few minutes, which is relatively fast [42]. The nongenomic effect mainly relies on the G protein-coupled estrogen receptor (GPER/GPR30), which was discovered in recent years. GPER, a member of the G protein-coupled receptor superfamily, is composed of 375 amino acids with a molecular weight of about 40 000 [43]. GPER is distributed in various organs and tissues, including breast, ovary, uterus, cardiovascular system, and lung and bone tissue, and is widely involved in the occurrence and development of estrogen-related diseases such as malignant tumors, inflammatory reactions, CVD, and obesity [44, 45]. The combination of E2 and GPER promotes the dissociation of the G protein trimer structure into *α*, *β*, and *γ* subunits [46]. The *α* subunit catalyzes cyclic adenosine monophosphate (cAMP) by activating adenylate cyclase on the cell membrane, and cAMP activates protein kinase A (PKA), thereby rapidly regulating cellular function changes [47–49]. In addition, *β* and *γ* subunits promote the release of heparin binding epidermal growth factor like growth factor (HBEGF) and the binding to epidermal growth factor receptor (EGFR), leading to the activation of multiple signal factors including mitogen activated protein kinase (MAPKs), phosphatidylinositol 3 kinase (PI3K), protein kinase B (PKB/Akt), and extracellular signal-regulated kinase (ERK1/2), which indirectly regulates the transcriptional activity of related genes and exerts various biological effects in the cell [47, 50–52] (Figure 1).

## 5. Oxidative Stress

The human body constantly produces oxygen free radicals during normal daily metabolic processes and approximately 95% of which are ROS, including superoxide anions (O_2_^−^), hydrogen peroxide (H_2_O_2_), hydroxyl free radicals (-OH), and peroxynitrite (ONOO^−^) [53]. Normally, the body's oxidation system and the antioxidant defense system maintain a dynamic balance. When the antioxidant and oxidative effects are out of balance, pathological damage occurs. This process is called OS [6]. The main sources of intracellular ROS include xanthine oxidase, lipoxygenases, cyclooxygenases, peroxidases, uncoupled nitric oxide (NO) synthases, NADPH, the mitochondrial respiratory chain, and heme-containing proteins, and among these, an abnormal mitochondrial respiratory chain is the main source of ROS [54]. When the mitochondria cannot undergo normal oxidative phosphorylation, many ROS are produced [55, 56]. The generated ROS damage organelles, such as the mitochondria and plasma membrane and the DNA, proteins, and lipids of the organelle components, which eventually leads to cell death, aggravate the production of mitochondrial ROS and form a vicious circle [57, 58]. Finally, tissue cell dysfunction, such as endothelial cell dysfunction, vasculitis, and the accumulation of low-density lipoprotein in the arterial wall, is triggered. In addition, ROS are not only potentially harmful products of metabolism [59]. They can also act as second messengers to regulate cell growth and apoptosis [60]. The intracellular antioxidant defense system includes superoxide dismutases (SOD), catalase (CAT), glutathione peroxidases (GPx), and other nonenzymatic antioxidants, such as reduced glutathione (GSH), vitamin C, vitamin E, *β*-carotene, ubiquinone, lipoic acid, and flavonoids, which can inhibit the formation of ROS or reduce the damage caused by ROS [61–63]. Therefore, new treatments involve not only eliminating ROS but also inhibiting the activity of ROS-generating enzymes.

There is an important relationship between sex and OS. Studies have demonstrated that male rats have a higher degree of OS than female rats [64]. Another in vivo study showed that young men have higher OS biochemical markers than women of the same age [3]. In addition, clinical and experimental data show that women have greater antioxidant potential than men [65]. In summary, there is a critical relationship between gender and OS [65, 66]. Women are not susceptible to OS and have stronger antioxidative stress capabilities than men, which further demonstrate that there is a strong connection between female estrogen and antioxidants [3].

## 6. Cardiovascular Diseases Mediated by OS after Menopause

After menopause, due to the exhaustion of ovarian follicles, the production of estrogen is greatly reduced, and the production of extraovarian estrogen becomes dominant [67]. During this period, the main plasma estrogen is estrone, which is less effective than E_2_ [68]. Premenopausal women have higher levels of NO, which protects the heart and inhibits smooth muscle proliferation in heart disease [38]. After menopause, due to the decrease in estradiol antioxidants, postmenopausal women are more likely to undergo OS than women of reproductive age, and the incidence of CVD increases [69]. Moreover, the significant reduction in estrogen increases the level of free fatty acids, which makes postmenopausal women more likely to develop metabolic syndrome and insulin resistance, which are considered risk factors for CVD [70, 71].

**Figure 2:**
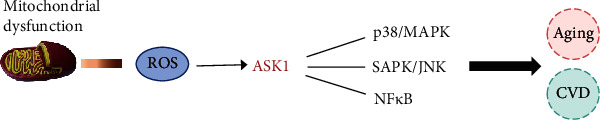
ROS promoted aging and cardiovascular disease by regulating ASK1.

OS is the main cause of many age-related cardiovascular pathologies, including ischemia/reperfusion (IR), hypertensive heart disease, and heart failure [72]. Under physiological conditions, low levels of ROS produced by mitochondria play an important role in vascular endothelial cells, which are involved in the production of NO, regulation of cell apoptosis, and signal transduction [73]. Some signaling pathways that promote aging, mainly including ASK1-p38-MAPK, ASK1-SAPK/JNK, and ASK1-NFkB, are involved in menopause [74]. These signaling pathways are also involved in oxidative stress-mediated CVD. The ASK1 signal body is a high-molecular weight protein complex composed of ROS-sensitive inhibitor protein and activator protein [74]. Its molecular weight is approximately 1500 kDa, which regulates the response to ROS and the signaling networks that promote aging and age-associated diseases of OS [74, 75]. The ROS related to aging mainly originate from mitochondrial dysfunction [76]. The generated ROS can activate the p38-MAPK and SAPK/JNK pathways, thereby mediating the occurrence of CVD [74]. In an aging mouse model, the inhibition of OS delayed aging through the p38-MAPK pathway, which means these signaling pathways have a certain relationship with aging, OS, and CVD [77] (Figure 2). The mechanism by which ROS mediate CVD is introduced below.

### 6.1. Oxidative Stress and Hypertrophic Cardiomyopathy (HCM)

HCM is characterized by left ventricular hypertrophy, a reduced ventricular cavity and limited ventricular filling [78, 79]. In HCM, Ca^2+^ in myocardial cells in combination with myofilaments can reduce the concentration of Ca^2+^ in the mitochondria, the activity of mitochondrial tricarboxylic acid cycle enzymes, and the level of reduced coenzyme I/II, thereby triggering OS [80]. In addition, excessive production of mitochondrial ROS leads to activation of Ca^2+^ channels and transporters in cardiomyocytes, which activates transcription factors related to cardiomyocyte hypertrophy [79]. Cardiomyocyte hypertrophy can lead to excessive production of mitochondrial ROS, and excessive ROS can cause cardiomyocyte hypertrophy, thus forming a vicious circle [81]. A previous study confirmed that E_2_ reduced myocardial OS and improved myocardial diastolic function, prevented myocardial energy dysregulation, thereby improving HCM [82].

### 6.2. Oxidative Stress and Atherosclerosis

Atherosclerosis is the leading cause of CVD [83]. Increasing evidence showed that the activation of proinflammatory signals, the expression of cytokines/chemokines, and OS are important factors leading to the occurrence of atherosclerosis [84]. Harmful stimuli (such as dyslipidemia, hypertension and smoking) can cause endothelial cell dysfunction, promote the expression of adhesion factors and chemotactic molecules, and increase the permeability of macromolecules [85]. This activity facilitates LDL entry into the arterial wall, resulting in apolipoprotein B100 and extracellular matrix (ECM) proteoglycan binding and retention [85]. In addition, oxidized low-density lipoprotein (OxLDL) activates endothelial cells to release phospholipids [86]. NOX2 is a specific subtype of NADPH oxidases (NOXs) and has been identified to play a key role in atherosclerosis formation [87]. NOX2 deficiency has little effect on blood lipids, but it can reduce the formation of aortic superoxide, increase the bioavailability of NO, and reduce the formation of atherosclerotic plaques [88]. Judkins et al. [89] found that in knockout apolipoprotein E (Apo E -/-) mice, the expression of NOX2 in mouse aortic endothelial cells and macrophages increases before atherosclerosis, and these changes are consistent with the increase in aortic superoxide production. Therefore, this study clearly showed that NOX2 plays a key role in superoxide generation, NO bioavailability reduction, and atherosclerotic plaque formation [90]. In conclusion, OS plays an important role in the progression of atherosclerosis. There is increasing evidence suggested that age is an important risk factor for atherosclerosis, which is promoted by cellular senescence [91]. E2 retarded oxidized low-density lipoprotein-induced premature senescence, thereby inhibiting arterial aging and the development of atherosclerosis [92].

### 6.3. Oxidative Stress and Heart Failure (HF)

HF is the terminal stage of heart disease. Many experiments and clinical studies have shown that ROS production is related to the pathogenesis of HF [93, 94]. By activating transcription factors and G-protein coupled receptors (GPCRs), ROS can stimulate myocardial cell growth and matrix remodeling and accelerate cell dysfunction [95]. The effects of H_2_O_2_ on adult rat ventricular myocytes are concentration-dependent [96, 97]. Low H_2_O_2_ concentrations can cause cardiomyocyte hypertrophy by activating ERK1/2, while high H_2_O_2_ concentrations can activate JNK and cause cardiomyocyte apoptosis [98]. ROS can also affect the extracellular matrix, stimulate the proliferation of cardiac fibroblasts, and activate matrix metalloproteinases (MMPs), which are the basic effects leading to fibrosis and matrix remodeling [99]. MMPs play an important role in the process of normal tissue remodeling, such as cell migration, invasion, proliferation, and apoptosis and have been shown to be elevated in HF [100]. MMPs are usually secreted in an inactive form and are activated by ROS after translation [100]. Hayashidani et al. [101] have shown that the survival rate of MMP-2 knockout mice after myocardial infarction (MI) is significantly improved because knocking out MMP-2 reduces the incidence of early heart rupture and left ventricular remodeling and failure. Kinugawa et al. [102] explored the role of OS in left ventricular remodeling and failure after myocardial infarction in mice and whether the -OH scavenger dimethylthiourea can alleviate these changes, and compared with untreated mice, mice who received dimethylthiourea demonstrated inhibition of MMP-2 activation, significantly improved left ventricular contractility, and reduced left ventricular hypertrophy. These findings indicate that OS products can stimulate the activation of myocardial MMP, and MMP plays a decisive role in left ventricular remodeling, thereby participating in the development of heart failure [103].

In recent years, OS markers, such as 8-OHdG, which has attracted much attention, have increasingly been used to assist in the diagnosis of heart failure [104]. These markers cause oxidative damage to DNA and serve as biomarkers of endogenous and exogenous factors [105]. The interaction between advanced glycation end products (AGEs) and their receptors (RAGE) initiates a series of signal cascade reactions, activating the transcription factor NF-*κ*B and leading to the release of inflammatory cytokines, such as tumor necrosis factor-*α* (TNF), and eventually inducing OS; so, AGEs and RAGE are considered OS markers [106]. Another marker, neopterin, is mainly produced by macrophages after *γ*-interferon stimulation [107]. The higher the neopterin concentration, the higher the NYHA heart function classification, and the higher the probability of CVD. In addition, neopterin is related to the formation of ROS. In summary, biomarkers of OS can be used as reliable indicators for the diagnosis of heart failure [95]. The development of HF is characterized by increased OS in cardiomyocytes. The increased production of ROS correlates with the progression of HF [108]. E2 treatment improves HF by antioxidative mechanisms, and E2 may be an effective adjunctive therapy for patients with HF [109].

### 6.4. Oxidative Stress and Hypertension

Hypertension is the most common chronic disease, and it is a major risk factor for CVD. OS is a contributing factor in the pathogenesis of hypertension [65]. Excessive OS and a weakened ability to scavenge free radicals can lead to hypertension. Although the sources of intracellular ROS are diverse, the activity of NOXs is the main source of ROS [110]. There are five subtypes of NOXs: NOX1, NOX2, NOX3, NOX4, and NOX5 [111]. In the vasculature, different cells and blood vessels express different NOX subtypes, and there is no specific NOX subtype. NOX4 is mainly expressed in endothelial cells and vascular smooth muscle cells [112]. NOX1 is mainly expressed in large blood vessels, while NOX2 is mainly expressed in resistance blood vessels [103]. In the vasculature, ROS is mainly produced by vascular endothelial cells, adventitia cells, and smooth muscle cells, and it is mainly the NADPH enzyme that produces O_2_- under the stimulation of angiotensin II (Ang II) and endothelin-1 [113]. The generated ROS can act as second messengers in the cell, increasing the intracellular concentration of Ca^2+^, causing vasoconstriction, thereby promoting the development of hypertension [114].

The endothelium is a type of highly active monolayer that plays an important role in regulating vascular wall tension, cell adhesion, thrombosis, smooth muscle cell proliferation, and vascular inflammation [115]. All of these roles are achieved by releasing endothelium-derived relaxing factors, such as prostaglandins, nitric oxide, endothelium-derived hyperpolarizing factors, and endothelium-derived contractile factors [1]. The generated vasodilating factor NO is rapidly degraded by the oxygen free radical O_2_-, and the superoxide anion produced by NOX reacts with NO to create peroxynitrite, which reduces the bioavailability of NO and causes vasoconstriction [103]. Therefore, hypertension is related to a decrease in NO and an increase in OS. Ang II is the main bioactive peptide of the renin-angiotensin system (RAS), which plays an important role in vasoconstriction, hypertrophy, fibrosis, inflammation, and aging [116]. Ang II activates the Ang II type 1 (AT_1_R) and type 2 (AT_2_R) receptors and drives OS through membrane-bound NADPH to increase the production of O_2_^−^ [117]. The mechanisms by which Ang II mediates its physiological and pathophysiological vascular effects are complex [116, 118]. Previous studies have shown that ROS production and activation of reduction-oxidation dependent signaling cascades play key roles in Ang II-induced actions [119]. ROS is produced by various types of vascular cells, including endothelial cells, smooth muscle cells, outer membrane fibroblasts, and resident macrophages [120]. The main source of ROS in vascular cells is nonphagocytic NADPH oxidase, which is regulated by vasoactive agents (including Ang II) [121]. Rajagopalan et al. [122] found that long-term infusion of Ang II increases the oxidase activity of NADPH so that hypertension can be reduced. Some common antihypertensive drugs, such as angiotensin-converting enzyme (ACE) inhibitors and angiotensin receptor inhibitors, can reduce blood pressure by inhibiting NOXs and reducing the production of ROS [120]. Hypertension susceptibility in women increases at the transition to menopause, and altered estrogen signaling is implicated in the increased hypertension incidence associated with menopause [123]. ER-*β* signaling plays an important role in blood pressure regulation. The inhibition of increased NMDA receptor signaling and ROS production in ER-*β* neurons in the paraventricular nucleus of the hypothalamus can reduce the susceptibility to hypertension [124].

### 6.5. Oxidative Stress and Atrial Fibrillation (AF)

AF is the most common arrhythmia in clinical settings. Many experiments have confirmed the role of OS in the pathogenesis of AF [125, 126]. By inducing cardiomyocyte hypertrophy and apoptosis, ROS have a destructive effect on calcium transport channels in cardiomyocytes, leading to arrhythmias and enhancing cardiac remodeling [127]. The atrial type 2 ryanodine receptor (RyR2) has been shown to be a target of OS and is involved in the pathogenesis of AF [128]. The abnormality of intracellular Ca2+ plays an important role in the occurrence of AF [128]. RyR2 is the main calcium release channel in atrial myocytes, and it can become dysfunctional due to OS [129]. The increased RyR2-dependent Ca2+ leakage due to enhanced CaMKII activity can increase the susceptibility of AF [128]. Thus, changing intracellular Ca^2+^ homeostasis is related to the pathogenesis of AF. Studies have shown that reducing the production of ROS can decrease the release of atrial Ca^2+^ during diastole, which hinders the development of AF [130]. Due to the low efficiency of DNA proofreading and repair, human mitochondrial DNA is prone to oxidative damage and mutation during replication. Lin et al. [131] speculated that increased OS and mitochondrial DNA mutation may be related to AF. Polymerase chain reaction (PCR) analysis showed that the probability of mitochondrial DNA deletion in the atrial muscle of patients with AF was 3.75 times higher than that of patients without AF, and the level of oxidative damage to DNA in patients with AF was also higher than that in patients without AF [131]. Dudley et al. [132] used a swine model of AF to further confirm that OS is related to the pathogenesis of AF. In addition, Bretler et al. [133] indicated that E_2_ therapy was associated with a decreased risk of new-onset AF especially among women ≥ 80 years old. E_2_ therapy can reduce the risk of AF by 9-37 percent, the first year after myocardial infarction.

### 6.6. Oxidative Stress and Ischemic Cardiomyopathy (ICM)

ICM refers to the clinical syndrome of chronic myocardial ischemia caused by coronary atherosclerosis, leading to diffuse fibrosis of myocardium and loss of myocardial function [134]. It is one of the most common causes of end-stage heart failure. Previous studies have shown that OS is closely related to the occurrence and development of ICM [135, 136]. During the pathogenesis of ICM, ischemia and hypoxia trigger a series of physiological and pathological processes, which make ROS accumulate in cells and promote OS [135]. The excessive production of ROS or the reduction of ROS clearance can damage the cell structure, destroy the cell membrane through lipid peroxidation, impair the function of enzymes through the oxidation of proteins, and cause chromosome damage through nucleic acid base modification and chain rupture, thus causing cell dysfunction [137]. In the process of ICM, ROS can destroy the cell membrane during the ICM process, promote calcium overload, cell apoptosis and the production of inflammatory mediators, and damage the function of endothelial cells and platelets, thereby promoting the occurrence and development of ICM [135, 138–141].

## 7. Estrogen Inhibits Oxidative Stress

OS is associated with a variety of diseases, including heart failure, hypertension, and atherosclerosis. Therefore, OS is an important mechanism of CVD, and any gender differences related to OS may affect the pathogenesis of CVD [3]. Estrogen may not be the only cause of gender differences between men and women but further research is needed to determine the protective effects of estrogen and the mechanisms involved. Jeanes et al. [142] found that E_2_ and ER*α*-specific agonists decreased the infarct size by reducing myocardial lipid peroxidation during I/R in rats. In a hypoxia and reoxygenation model of rat cardiomyocytes in vitro, E_2_ reduced cardiomyocyte apoptosis and ROS production by decreasing MAPK activity [143]. Estrogen decreases the risk of CVD by downregulating inflammatory markers, such as chemokines and cell adhesion molecules, to fight atherosclerosis [144]. In addition, it can stabilize atherosclerotic plaques by reducing the expression of matrix metalloproteinases and the production of plasminogen activator inhibitor-1 (PAI-1) [145]. Moreover, high concentrations of estrogen promote vasodilation by producing prostacyclin, inhibiting endothelin synthesis and blocking calcium channels [1]. In addition to its benefits to the cardiovascular system, estrogen also has an effect on biomarkers of vascular activity [146]. For example, a study concerning normal postmenopausal women revealed that taking estrogen for one year significantly reduced catecholamine levels, mean blood pressure, and low-density lipoprotein (LDL), while increasing nitrite and nitrate levels [147–149]. Other studies on the effects of estrogen on OS have shown that serum lipid peroxides decrease, and the overall antioxidant status is upregulated [150]. Estrogen increases binding proteins produced by the liver, such as sex hormone binding globulin, water maintenance, and sodium balance in the body, and it distributes lipids by increasing high-density lipoprotein (HDL) and reducing LDL [151]. It is clear that there is a definite relationship between estrogen and OS (as shown in Table 1).

Some studies have shown that estrogen inhibits OS in cardiac vessels and the myocardium by reducing local ROS production and increasing ROS clearance [143]. In addition, the removal of ROS in the blood vessel wall and heart is essential to ensure the structural and functional integrity of the cardiovascular system. NOXs is the main source of ROS [152]. Estrogen regulates the expression of NOXs subunits in different models, which has a protective effect on the cardiovascular system [153]. NOXs is an oxidase complex composed of NOX1-5, dual oxidase, and regulatory subunits p22^phox^, p47^phox^, p67^phox^, p40^phox^, and Racl [111, 154]. Supplementation of E_2_ in ovariectomized rats inhibited the reduction of superoxide anion production by the NOX subunit p47^phox^ [155]. This finding suggested that estrogen changes the production of superoxide anions by regulating the expression or activity of NOXs in vascular smooth muscle cells [156]. Ang II can increase the expression of Rac1 protein in vascular smooth muscle cells, while E_2_ can restore it to normal levels [157]. Zhang et al. [158] found that the expression of p22^phox^ increased in salt-sensitive ovariectomized rats that were fed a high-sodium diet, which was reversed by injection of estrogen. Estrogen can also reduce the expression of the NOXs subunit NOX2 in endothelial cells in a time- and concentration-dependent manner, and this effect can be blocked by ER antagonists [159]. To summarize, both estrogen deficiency and estrogen supplementation change the expression and activity of NOXs, thus changing the production of O_2_^−^ [160]. However, due to the different regulation of NOXs subunits in different animals and cells, it is not completely clear how estrogen affects the activity of NOXs through complex mechanisms (as shown in Table 1).

The renin-angiotensin-aldosterone system (RAAS) is an important humoral regulatory system composed of some peptide hormones and corresponding enzymes, which mainly maintain and regulate the balance of blood pressure, water, and electrolytes and maintain human homeostasis [161]. In vivo and in vitro studies have demonstrated that the RAAS plays a key role in the pathogenesis of CVD [162]. Ang II activates the AT_1_R and mediates most of the biological effects of Ang II, such as vasoconstriction, aldosterone release, sodium and water maintenance, and cell growth. AT_1_R-related NOXs produce many highly active O_2_^−^ molecules, which are the main source of RAAS-induced ROS production, in monocytes, macrophages, endothelial cells, and vascular smooth muscle cells [163]. In addition, estrogen deficiency can also increase the expression of the angiotensin converting enzyme (ACE), thus promoting the production of Ang II [164]. Nickenig et al. [165] found that estrogen deficiency can upregulate AT_1_R in isolated vascular smooth muscle, while estrogen supplementation can reverse this phenomenon. The expression of NOXs subunits gp91^phox^, p22^phox^, and p67^phox^ induced by Ang II are decreased by E_2_ [143] (as shown in Table 1).

SOD, which converts O_2_^−^ into H_2_O_2_, is a cellular antioxidant defense mechanism and has been shown to be regulated by steroids [166]. Strehlow et al. [167] found that E_2_ upregulated the expression and activity of SOD in vascular smooth muscle cells induced by Ang II, thus inhibiting the production of ROS induced by angiotensin converting enzyme II. In ovariectomized rats, the expression of antioxidant enzymes GPX1 and GPX4 significantly decreased, but estrogen returned expression to normal values [158]. Estrogen can also increase the expression of the glutathione rate-limiting enzyme *γ*-glutamylcysteine synthetase, which is consistent with the activation of glutathione reductase promoter activity by ER*β*-specific *cis*-acting elements [168] (as shown in Table 1).

Nitric oxide synthase (eNOS) produced by endothelial cells can produce the vasodilator NO, and NO spreads to vascular smooth muscle cells, activates guanylate cyclase, and increases cyclic guanosine monophosphate (cGMP) [84]. NO plays a direct role in tissue oxygen balance, organ perfusion, vascular remodeling, and metabolic requirements by regulating vascular tension and diameter [169]. Kauser et al. [170] first found that there were gender differences in the production and release of NO, and the release of NO in the aorta of female rats was higher than that of males. Estrogen maintains the bioavailability of NO by increasing the expression of eNOS mRNA and protein, thus increasing the production of NO in endothelial cells and vascular smooth muscle cells [171]. Wassmann et al. [172] demonstrated that raloxifene, a selective estrogen receptor modulator, increases the bioavailability of NO by upregulating eNOS mRNA and activity in the aorta of spontaneously hypertensive rats. Estrogen deficiency can increase blood pressure, produce OS, and decrease NO production. Similarly, estrogen supplementation increased NO production and decreased the amount of lipid peroxidation in ovariectomized rats [173]. However, Barbacanne et al. [174] believe that the antioxidant effects of estrogen are not achieved by affecting the activity or expression of eNOS but by directly decreasing the production of O_2_^−^. In vivo and in vitro experiments demonstrated that estrogen produces NO through nongenomic effects, and the specific mechanism is that ER*α* activates eNOS through the PI3/AKT signal pathway to produce NO. [175] Wong et al. [176] confirmed that raloxifene can increase the phosphorylation of eNOS and Akt in rat aortae and protect endocrine cells from OS. Estrogen can also increase the intracellular availability of the eNOS cofactor BH4 and prevent the uncoupling of eNOS, thus preventing the production of eNOS-dependent ROS [177] (as shown in Table 1). To summarize, estrogen can be used as a potential mechanism of antioxidation by increasing the production of NO, reducing O_2_^−^, and increasing the utilization of cofactor BH4 [178]. To support this hypothesis, postmenopausal women taking BH4 can improve endothelial dysfunction and reduce the incidence of atherosclerosis [179].

## 8. Conclusion

The incidence of CVD is lower in premenopausal women than in men of the same age, but it significantly increases after menopause. This phenomenon shows that estrogen has a protective effect on the cardiovascular system, which is undeniable. OS is an important mechanism of cardiovascular disease. This article mainly indicates the protection of estrogen in cardiovascular disease from the perspective of OS. When postmenopausal women are treated with estrogen, a comprehensive assessment should be performed according to the patient's symptoms, CVD and breast cancer risk, etc. to determine the route of administration, dosage, and frequency, and the risks and benefits should be regularly assessed to obtain minimal risk and maximal benefit through individualized treatment.

## Figures and Tables

**Table 1:** The mechanisms of estrogen inhibiting oxidative stress.

Mechanisms	The changes in oxidative stress	References
E_2_ decreased MAPK activity	The cardiomyocyte apoptosis and ROS production were reduced	[74, 77, 143, 180]
Estrogen decreased serum lipid peroxides	Overall antioxidant status was upregulated	[92, 150, 173, 181]
E_2_ inhibited NOX subunit p47phox	The reduction of superoxide anion production was inhibited	[155, 160]
E_2_ decreased NOX subunits gp91^phox^, p22^phox^, and p67^phox^ induced by Ang II	ROS production was reduced	[143, 158, 182, 183]
E_2_ upregulated the expression and activity of SOD induced by Ang II	ROS production wad reduced	[167, 184–190]
Estrogen restored antioxidant enzymes GPX1 and GPX4 expression levels	Oxidative stress balance was maintained	[158, 181, 189]
Estrogen increased the expression of the glutathione rate-limiting enzyme *γ*-glutamylcysteine synthetase	Oxidative stress balance was maintained	[168, 190, 191]
Estrogen maintained the bioavailability of NO by increasing the expression of eNOS mRNA and protein	The production of NO increased and oxidative stress was reduced	[84, 192–195]
ER*α* activated eNOS through the PI3/AKT signal pathway	The production of NO increased and oxidative stress was reduced	[175, 189, 195]
Estrogen increased the intracellular availability of the eNOS cofactor BH4 and prevented the uncoupling of eNOS	The production of eNOS-dependent ROS was reduced	[177, 178]
